# Management of SARS-CoV-2 Prevention Methods in Dental Offices—A Cross-Sectional Study in Bucharest, Romania

**DOI:** 10.3390/healthcare12121169

**Published:** 2024-06-10

**Authors:** Mihnea Ioan Nicolescu, Oana Irina Lupu, Raluca Ștefania Georgescu, Mihai Săndulescu, Cristian Funieru

**Affiliations:** 1 Division of Histology, Faculty of Dentistry, “Carol Davila” University of Medicine and Pharmacy, 050474 Bucharest, Romania; 2 Laboratory of Radiobiology, “Victor Babeș” National Institute of Pathology, 050096 Bucharest, Romania; 3 Endodontics Residency Department, “Sf. Pantelimon” Clinical Emergency Hospital, 021659 Bucharest, Romania; oana.lupu@prof.utm.ro; 4 Independent Researcher, 011981 Bucharest, Romania; contact@ralucageorgescu.doctor; 5 Division of Implant Prosthetic Therapy, Faculty of Dentistry, Carol Davila University of Medicine and Pharmacy, 010221 Bucharest, Romania; mihai.sandulescu@umfcd.ro; 6 Division of Preventive Dentistry, Faculty of Dentistry, “Carol Davila” University of Medicine and Pharmacy, 050037 Bucharest, Romania; cristian.funieru@umfcd.ro

**Keywords:** COVID-19 pandemic, PPE, preventive dentistry, digital questionnaire

## Abstract

We compared the managing of prevention methods for SARS-CoV-2 infections in dental offices before and immediately after the COVID-19 pandemic. The purpose of this study was to find out the varieties of infection prevention methods used by dentists before and during the pandemic and compare them. We designed a digital transversal questionnaire with 15 closed questions that was sent to 150 dentists in Bucharest, Romania. We received *n* = 112 valid answers during July-August 2021 from dentists of all age groups (25–60 years), with a sex ratio of 0.36, which agreed to anonymously participate in this study. The results showed an increase in types and amount of personal protection equipment (i.e., ocular/facial protection, supplemental gown, and upgrading the FFP1 masks to FFP2 or FFP3). Ocular protection showed statistical significance by gender but not by age group. Vaccination rate against SARS-CoV-2 was at 80% of the participant dentists at the time of the survey and had statistical significance. However, vaccination status of the patients did not alter dentists’ protection protocol.

## 1. Introduction

The pandemic is defined as an epidemic that spreads over a large area, over a very large territory, and can reach the global level. Pandemics have been very important throughout the current era, having a huge impact on the population both physically and mentally, and resulting in millions of deaths [1].

Coronaviruses were first reported in late 1930 [2], causing various diseases in wild and domestic animals, for example bronchitis in chickens, enterocolitis in pigs, and hepatitis and neurological disorders in mice. Later, after these types of viruses were observed under an electron microscope [3], they were grouped into a family of viruses called Coronaviridae due to their appearance resembling the form of a crown. So far, seven human coronaviruses have been reported [4]. Cases of coronavirus disease recur every year, especially in the cold season. The following three types, SARS-CoV, MERS CoV, and SARS-CoV-2, are the cause of severe respiratory infections. The first case of SARS-CoV (Severe Acute Respiratory Syndrome) was discovered in 2002 in China. With patient 0 (the first infected patient) being known for certain, the epidemiological investigation was very well kept under control, so that in 6 months only 6000 cases were reported globally [5]. In 2012, 10 years later, the first patient infected with MERS CoV (Middle East Respiratory Syndrome) appeared in Saudi Arabia. The virus was transmitted to humans by camels and then spread by humans throughout the world. Human-to-human transmission is most common in hospitals. By 2019, 861 deaths were reported globally, with a mortality rate of 35% [1]. At the end of 2019, the first cases of pneumonia of unknown etiology were reported in Wuhan, China. Subsequently, on 7 January 2020, the etiology of pneumonia was discovered, namely the infection with the SARS-CoV-2 virus, and the triggered disease was named COVID-19 [6]. As of 27 February 2020, cases were reported in 47 countries with a total of 2804 deaths [7]. About a year after the virus began, preliminary estimates suggested the total number of deaths attributable to the COVID-19 pandemic in 2020 was at least 3 million [8].

The nationwide lockdown significantly reduced access to dental care. Moreover, supplemental regulations were put into place, many of them specifically addressing dental offices’ activity. The aim of this study was to compare the infection prevention methods used by dentists before and during the pandemic in the city of Bucharest, Romania by use of a digital questionnaire.

## 2. Materials and Methods

### 2.1. Ethical Aspects

This study was conducted in accordance with the Declaration of Helsinki and approved by the Institutional Review Board of the Faculty of Dentistry, “Carol Davila” University of Medicine and Pharmacy (#11/9 January 2021).

### 2.2. Study Design and Sample

A cross-sectional study was carried out with a sample of *n* = 112 participants, all qualified dentists in Bucharest, Romania. The participants were part of a larger group (*n* = 150) of dentists, selected randomly from a list of previously collected professional emails, representing about 3% of dentists in the specified area.

A 15 item closed questions digital questionnaire was designed, and it was accessible using a specific web link collector. The editing of answers was permitted until the participant finalized the questionnaire. Multiple access from the same device was restricted. The design and management of questions and answers were performed using a professional survey software platform: surveymonkey.com (SurveyMonkey Inc., San Mateo, CA, USA), using an Advantage License.

### 2.3. Eligibility Criteria

The following inclusion criteria were considered:

- Individuals practicing dentistry at the time of study in dental offices in Bucharest, Romania;
- Individuals practicing dentistry before and during the SARS-CoV-2 Pandemic;
- Individuals between 25 and 60 years old.

The following exclusion criteria were considered:

- Multiple access of the digital questionnaire from the same device;
- Dental offices closed after the debut of the pandemic (even after it was lawfully permitted).

### 2.4. Data Collection

Data was carried out through a structured digital questionnaire between July and August 2021, all the answers were anonymous, and the participants expressed their agreement to participate. It was assumed that the initial random selection of the pool, the personal will of participation, followed by the informed agreement constituted a sufficient element of chance in selecting the sample, hence we consider it random.

### 2.5. Statistical Analysis

Data analysis was performed using SurveyMonkey internal analysis functions and the SPSS processor, version 16 (SPSS Inc., Chicago, IL, USA). Statistical significance was considered if *p* < 0.05. A Chi-square test and a Fisher’s test were used.

### 2.6. Study Limitations

Limitations of this study include the low number of participants, the restricted geographical area and the various PPE options that each dental office provided for their doctors. The specificity of the questions included in our digital questionnaire were not optimized for conventional reliability, hence the Cronbach’s Alpha cannot be applied. The internal validity of the questionnaire was assessed by the authors on a small-sample test group, which was not included in the final pool of respondents

## 3. Results

### 3.1. Age and Gender

**Figure 1:**
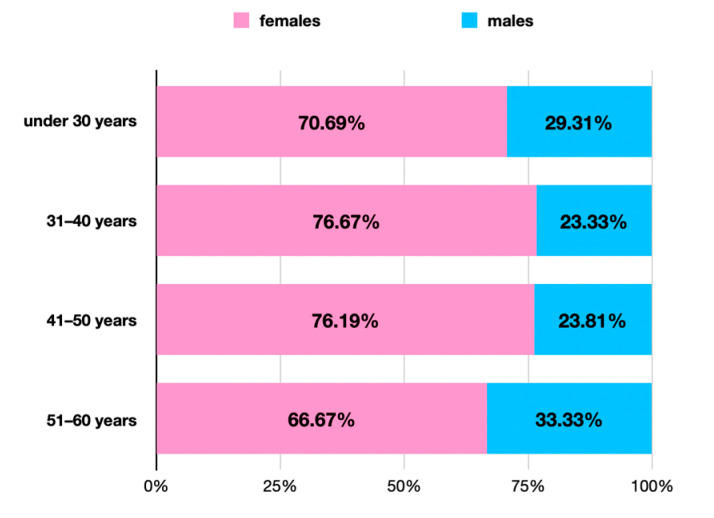
Gender distribution on age groups of participants.

Most of the responders (51.79%) were under 30 years old (y.o.), while 26.79% were between 31 and 40 y.o. Older age groups were less represented—the 41–50 y.o. accounted for 18.75% while only 2.68% were above 51 y.o. The participants identified as women (73.21%) and men (26.79%), yielding a sex ratio of 0.36. All the age groups had participants of both genders (Figure 1).

### 3.2. Usage of Surgical Mask before and during Pandemic

**Figure 2:**
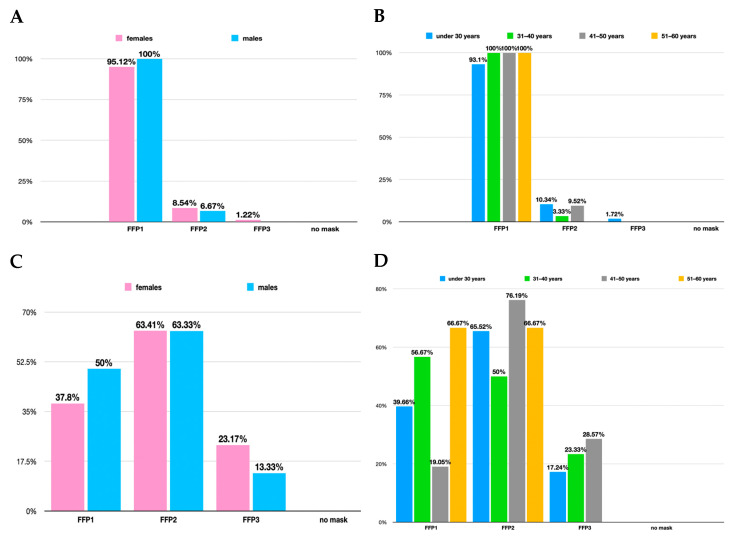
Type of mask used before (top row) and during (bottom row) SARS-CoV-2 pandemic, split on gender (**A**,**C**) and age group (**B**,**D**) distribution. Note: multiple choices were allowed.

Before the SARS-CoV-2 virus pandemic, 91.07% of the participating dentists wore a surgical mask during each intervention, while 6.25% wore a mask only during aerosol-generating maneuvers (AGM)—sonic descaling, airflow a.s.o.—or when the patient declared themselves infectious (1.79%). No answer was registered in the “never” category, and 0.89% declared the wearing of a surgical mask as “occasional”. As for the choice of the type of mask(s) that doctors used to wear before the pandemic with the SARS-CoV-2 virus, almost all (96.43%) used the FFP1-type mask (regular surgical mask), while the FFP2-type mask (the equivalent N95 masks) and the FFP3-type mask (solid and liquid aerosol blocking mask) were rarely used (8.04% and 0.89%, respectively). After splitting this result by gender and age groups (Figure 2 top row), no special preference for a certain type of mask was identified. Numerical values were allotted to the mask choices: 1 for FFP1, 2 for FFP2, and 3 for FFP3. The following results were obtained: 1.10 ± 0.34 for women and 1.06 ± 0.24 for men; in comparison to the overall mean of 1.09 ± 0.32 (using standard deviation and methodology from SurveyMonkey primary statistics package).

After the start of the SARS-CoV-2 pandemic, the situation changed. A total of 63.39% stated they used FFP2/N95 masks, a little over a fifth (20.54%) were using FFP3 masks, while the common surgical mask (FFP1) was still reportedly being used by 41.07% of the responders. This time, the weighted means using the same algorithm described above were 1.88 ± 0.69 for women and 1.71 ± 0.65 for men in comparison to the overall mean of 1.84 ± 0.68. That results in an overall increase in the protection indicator of 68.81% (from 1.09 to 1.84). The median value also increased from 1 to 2 (implying a shift in the preferred mask from FFP1 to FFP2/N95). The *χ*^2^ showed a weak significance when analyzing the age group differences (*p* = 0.06).

**Figure 3:**
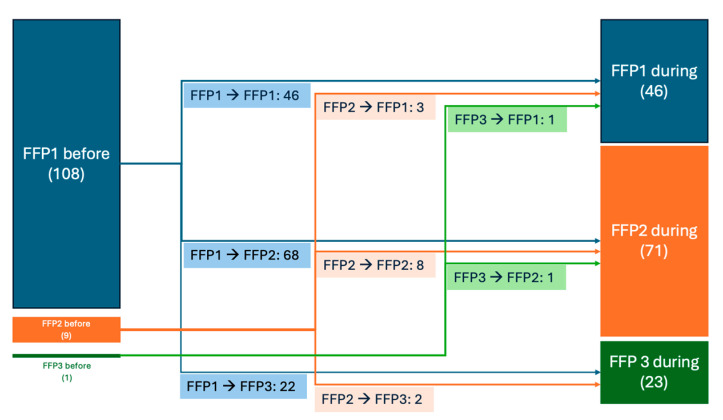
Change in mask type used before and during SARS-CoV-2 pandemic. (absolute numbers). Note: multiple choices were allowed.

The change in the mask type used before and during the SARS-CoV-2 pandemic was analyzed for each respondent, who were allowed to select more than one type of mask in their answers (Figure 3). Thus, out of initial users of FFP1 masks, 42.59% were still using it, while 62.96% upgraded to using FFP2/N95 and 20.37% to using FFP3 masks during the pandemic. On the other hand, of the dentists who previously wore FFP2/N95 masks before the pandemic, one third were using FFP1 masks, 88.89% were still using FFP2/N95 masks, and 22.22% had switched to FFP3 masks. As a peculiar detail, the single user of FFP3 before the pandemic switched to the use of FFP1 and FFP2 instead.

### 3.3. Usage of Ocular/Facial Protection before and during Pandemic

**Figure 4:**
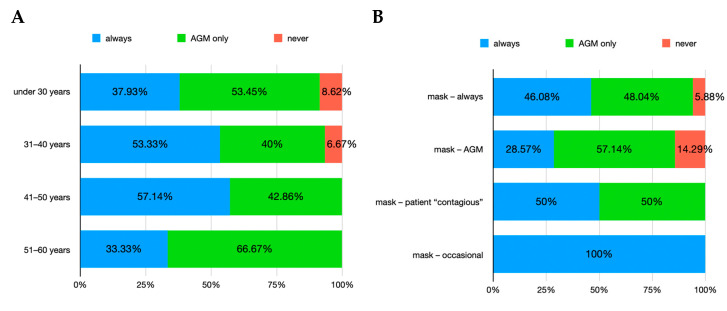
Usage of ocular/facial PPE before SARS-CoV-2 pandemic. (**A**) age group distribution; (**B**) relation with situations when mask was also worn. Note: AGM = aerosol-generating maneuvers; “patient contagious” = patient who self-declared a risk of contagion (e.g., hepatitis B).

For the question regarding the choice of wearing ocular/facial personal protection equipment (PPE) before the SARS-CoV-2 pandemic, the *χ*^2^ test showed statistical significance for gender (*p* = 0.008) but not for age groups (*p* = 0.82). The answers indicated that almost a half of the interviewed dentists were always using ocular/facial PPE (45.12% of women and 46.67% of men), while the other half only did so when they considered that their maneuvers were generating aerosols (51.22% of women and 40% of men). A few responders stated they were not using ocular/facial PPE at all (3.66% of women and 13.33% of men). When splitting the result by age groups, it has been noted that the percentage of dentists who always wore ocular/facial PPE before the pandemic varied between 33% and 57%, while those who wore them only during AGM ranged between 40% and 66% (Figure 4A). After cross-analyzing these answers with the situations when the same dentists declared they used the mask (Figure 4B), some dentists were always using both mask and ocular/facial PPE while others alternated the usage depending on the AGM or when patients declared themselves with a “contagious risk” (i.e., hepatitis B).

After the debut of the pandemic, 63.39% of dentists (70.73% of women and 43.33% of men) increased the frequency of ocular/facial PPE, while the remaining 36.61% (29.27% of women and 56.67% of men) used it with the same frequency.

### 3.4. Usage of Disposable Garment before and during Pandemic

**Figure 5:**
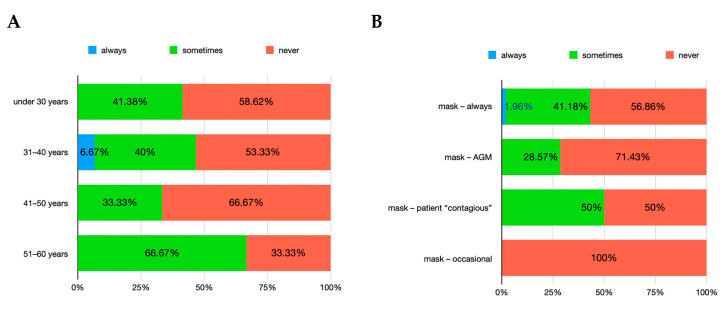
Usage of disposable garment over the surgical gown before SARS-CoV-2 pandemic. (**A**) age group distribution; (**B**) relation with situations when mask was also worn. Note: AGM = aerosol-generating maneuvers; “patient contagious” = patient who self-declared a risk of contagion (e.g., hepatitis B).

In regard to the usage of disposable garments over the surgical gown, before the pandemic, 1.79% of the interviewed dentists wore it always, while 40.18% wore it seldom and the majority (58.04%) never wore it. After breaking down this result by dentists’ responses to the question of whether they used to use a mask before the SARS-CoV-2 virus pandemic was declared, the dentists always wearing a mask were the same as those who also used a disposable garment over the surgical gown (Figure 5).

**Figure 6:**
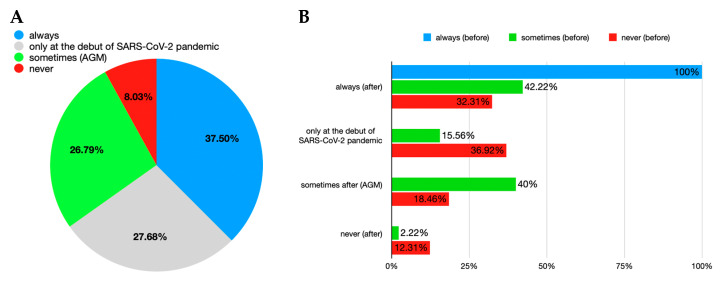
Usage of disposable garment over the surgical gown during SARS-CoV-2 pandemic. (**A**) overall; (**B**) relation with the answers regarding the pre-pandemic situation. Note: AGM = aerosol-generating maneuvers.

The start of the SARS-CoV-2 pandemic changed the attitude of the interviewed dental professionals, and now over a third are always wearing a supplemental disposable garment (Figure 6A). This result was then further interpreted in connection to the responses to the question about the frequency of use of disposable gowns/overalls over the medical suit by dentists prior to the declaration of the SARS-CoV-2 virus pandemic. Thus, all the doctors who previously used it still did after the declaration of the pandemic. Dentists who only used it sometimes had partially switched to regular use, as had some of those who never used it (Figure 6B).

### 3.5. Supplemental Actions in the Dental Offices during the Pandemic

More than 90% of the dentists used epidemiological questionnaires for patients, while only 3% were not using it at the time of this study and 4% declared they used it sometimes. Regarding the testing of each patient upon entering the dental practice for SARS-CoV-2 virus infection, 2.68% of the dentists tested all their patients (rapid antigen test/PCR), 79.46% did not test them, 10.71% of the doctors tested them only sometimes, and 7.14% only if the patient requested.

**Figure 7:**
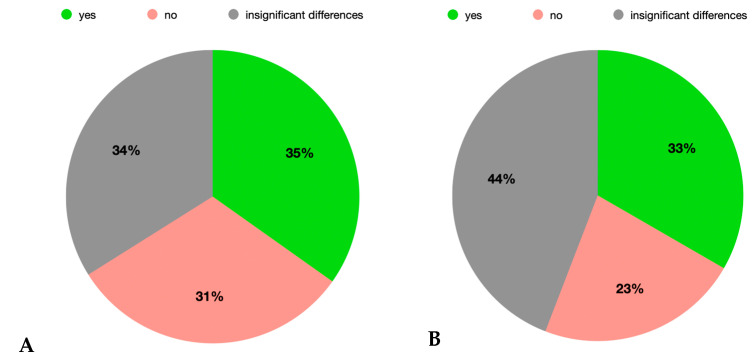
Dentists’ own assessment regarding the increase in time for washing hands (**A**) and of thoroughness for surfaces disinfection (**B**) after the debut of the SARS-CoV-2 pandemic.

It was noted that the answers were divide almost equally between yes, no, and “insignificant differences” in the questions about the time increase in washing hands (Figure 7A) and thoroughness of surface disinfection (Figure 7B) during the pandemic. However, when cross analyzing these two questions, the following results emerged: from the total of those who paid more attention to surface decontamination, almost three quarters allocated more time to hand washing. Of the total of those who did not pay an increased attention to the decontamination of surfaces after the debut of the pandemic, 16% allocated more time to hand washing, 80% did not allocate more time to hand washing, and 4% considered that the differences were insignificant. From the total of those who considered that the differences were insignificant regarding the greater attention paid to decontamination of surfaces, 16.33% allocated more time to hand washing, 24.49% did not allocate more time to hand washing, and 59.18% considered that the differences were insignificant.

### 3.6. Vaccination Status of Medical Professionals

**Figure 8:**
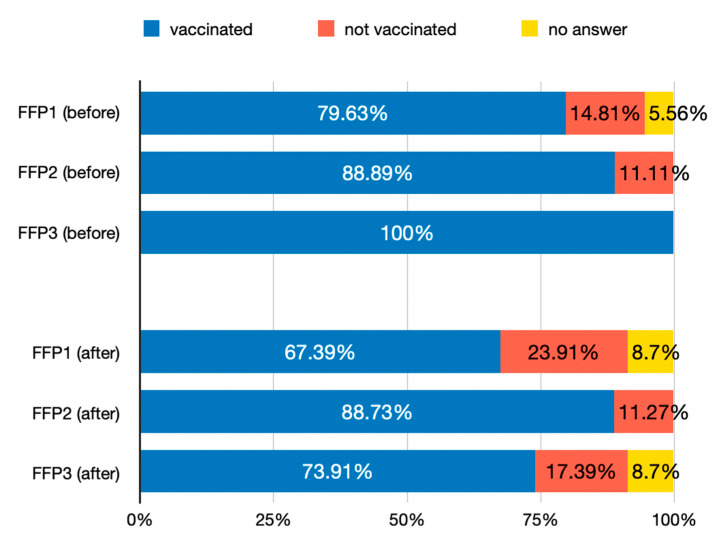
Vaccination against SARS-CoV-2 status among the interviewed dentists (August 2021) cross-analyzed with the type of mask worn before (**top**) and after (**bottom**) the debut of the SARS-CoV-2 pandemic.

The vaccination rate among the interviewed medical professionals at the time of this study (August 2021) was over 80% (14% unvaccinated and 6% chose not to answer). When cross-analyzing the answers to this question with some of the answers in earlier sections of the questionnaire, the results were split according to the type of masks the dentists used to wear before and after the debut of the SARS-CoV-2 pandemic (Figure 8). The Fisher’s exact test showed statistical significance for gender (*p* = 0.03) and weak significance for age groups (*p* = 0.059).

## 4. Discussion

The analysis of the answers to the digital questionnaire showed an increase in the methods and time allocated to the prevention of infections that can be transmitted in the dental office. Before the debut of the SARS-CoV-2 pandemic, most dentists (91.07%) protected themselves by wearing a mask during each intervention. Also, before the start of the pandemic, a large majority (96.43%) protected themselves by wearing the FFP1-type mask, normal surgical mask, which was changed after the pandemic debut by most dentists (63.39%) to the FFP2-type mask, the equivalent of the N95 mask, which had a greater potential to defend against the virus. A considerable part of them (20.54%) chose the FFP3-type mask—solid and liquid aerosol blocking mask—compared to the situation before pandemic, when this type of mask was only used by a small percentage of dentists (0.82%). An interesting aspect is represented by the great majority (62.96%) of all those who previously wore the normal surgical mask (FFP1) switching to wearing the FFP2/N95-type mask. It is worrying that some (33.33%) of the doctors who used to wear the FFP2/N95-type mask before, still included the FFP1-type mask on their list after the debut of the pandemic.

Previous studies revealed an increased contamination risk for AGM [9,10,11]. Most dentists (48.21%) wore eye/face protection sometimes, when they considered the maneuvers to be aerosol generators, before the pandemic. The situation improved during the pandemic, and 63.39% indicated a more frequent use of eye/face protection.

It has been recently published that a full mask (even protecting the doctor’s neck) should be worn [12]. Wearing the disposable gown/overall before the pandemic was not an option for the vast majority (58.04%) in our study before the pandemic, and this changed to 37.50% of them who used it all the time. From the total number of doctors who used to wear the mask throughout the intervention before the declaration of the pandemic, the great majority of them (48.04%) wore eye/face protection only sometimes, when the maneuvers were generating aerosols, but the great majority (56.86%) did not used to wear a disposable gown/overall over the medical suit. At the same time, from the total number of doctors who used to wear the mask throughout the intervention before the pandemic, after its debut, the vast majority (62.75%) wore eye/face protection more often and disposable gown/overalls all the time (37.25%).

While other studies reported that many dental offices chose to close during pandemic [13], the responders in our study were all maintaining their clinics as open to the public. Most dentists (92.86%) participating in this study applied the epidemiological triage questionnaire to each patient upon entering the dental office but did not test each patient (79.46%). Thus, most doctors who applied the questionnaire to each patient, (68.27%) used eye/facial protection more often, (40.38%) wore a disposable gown/overall over the medical suit and protected themselves using the FFP2/N95-type mask (66.35%) after the debut of the pandemic. A large percentage of dentists (44.14%) considered the differences to be not significant enough to pay more attention to the decontamination of surfaces than they did before the pandemic but allocated more time to hand washing (34.83%).

Following the pandemic, most respondents to our questionnaire (80.36%) declared themselves vaccinated against SARS-CoV-2 infection. This is in accordance with previously published data [14,15,16]. Among them, almost all (96.67%) applied the questionnaire for epidemiological triage to each patient, but 8 out of 10 did not test the patient upon entering the office. Also, 3 out of 10 believed that they paid more attention to the decontamination of surfaces than they did before, and 4 out of 10 believed that they spent more time washing their hands.

An important aspect is represented by the fact that, among unvaccinated doctors, the vast majority (68.75%) used the FFP1-type mask (ordinary surgical mask), they did not use eye/facial protection more often (with the mention that 7 out of 10 stated that they used eye/face protection all the time before the pandemic), but they tended to wear disposable gown/coveralls (31.25%) after the debut of the pandemic.

The dynamics of private dental offices in Romania are very important since the level of public finances of dental medical services are below the average of the EU [17]. Moreover, we must account for the importance of good communication, especially during the pandemic, between health providing professionals and the local and national authorities [18,19,20,21]. As published shortly after the pandemic [22], one of the earliest measures in trying to limit the spread of the pandemic outcome was the issue of guidelines to be adopted by dentists while treating their patients [20,21,23]. All the measures, as well as the associated costs, leave their mark on the economical side of any dental private practice [24]. The future will be different as well, from a broader integration of biotelemetry [25] and telemedicine into the public health response (even dentistry—as pointed out in a recent report [26]) to changes in dental practices and schools of dental medicine worldwide [27]. One of the easiest elements would be the inclusion of a careful telephone screening for patients before their schedule at a dental clinic, to minimize the risk of spreading (any) transmissible disease [28], as other medical specialties are also proposing [29].

Another factor that other studies are mentioning is the ventilation (quantitively and qualitatively), and it would be interesting to compare it before [30] and after [31] the pandemic; however, we have not included this in our current approach since we considered that there is a high variation of dental offices room types in terms of area, volume, and glazed surface.

Although different procedures depending on the assessed infectious risk were proposed [32], we consider that proper measures should be in place for all patients, especially considering the dentists’ risk of exposure to bodily fluids [33,34]. Hence, the role of personal protective equipment is pivotal, not only during the pandemic [35] but in the post-pandemic era as well [36].

## 5. Conclusions

The prevention of transmissible infections in the dental office is a matter of general interest, especially after the debut of the SARS-CoV-2 pandemic. Thus, dentists have increased/diversified the prevention methods they were using, for the safety of both patients and the entire medical staff. This study sought to find out the infection prevention methods that dentists used before the SARS-CoV-2 pandemic and compare them with those used after the pandemic debut using a digital questionnaire that allowed cross-analyzing answers to various sections.

### 5.1. Changes in PPE Type/Usage

As expected, a high number of dentists were using surgical masks during every intervention, before the pandemic was declared. However, a large percentage of dentists who used to wear the FFP1-type mask (ordinary surgical mask), replaced it with the FFP2-type mask (equivalent to an N95 mask) or FFP3-type mask (solid and liquid aerosol blocking mask) after the declaration of the pandemic. Dentists who did not always wear eye/face protection before the pandemic stated that after the pandemic, they used it more often (the results showed strong statistical significance when gender-analyzed). An interesting result was that after the declaration of the pandemic, more doctors wore the disposable gown/overall over the medical suit.

### 5.2. Supplemental Measures in Dental Offices

Nine out of ten dentists applied the epidemiological triage questionnaire to each patient at the moment of study (August 2021). However, testing the patient upon entering the dental office was not a priority for the dentists included in our survey. Dentists spent more time washing their hands than they did before the SARS-CoV-2 pandemic was declared. Due to the modified epidemiological situation, after the declaration of the SARS-CoV-2 pandemic, dentists have paid more attention to infection prevention measures in the dental office. Thus, increasing attention to the prevention of infections has, and may continue to have, a beneficial effect in the management of the epidemiological situation. Vaccination has been taken seriously by the dental medical staff, as 8 out of 10 doctors declared having been vaccinated against the SARS-CoV-2 virus, a result that showed significance both for age and gender.

Future studies should analyze the effects of the increased attention of dentists on infection prevention measures after the declaration of the SARS-CoV-2 pandemic, reflected in the possible variations in the incidence of infections with different viruses mentioned previously.

## Data Availability

The data presented in this study are available upon request from the corresponding author.

## References

[1] Dattani S. What Were the Death Tolls from Pandemics in History?. https://ourworldindata.org/historical-pandemics.

[2] Beaudette F.R., Hudson C.B. (1937). Cultivation of the virus of infectious bronchitis. J. Am. Vet. Med. Assoc..

[3] Almeida J.D., Tyrrell D.A. (1967). The morphology of three previously uncharacterized human respiratory viruses that grow in organ culture. J. Gen. Virol..

[4] Poutanen S.M. (2018). Human Coronaviruses. Principles and Practice of Pediatric Infectious Diseases.

[5] Ramaekers K. General perspectives from the virologists point of view. Laboratory of Clinical & Epidemiological Virology. Proceedings of the Oral Health Research Congress, Young CED-IADR Webinar, The Impact of COVID-19 on Oral Health, COVID-19 and Oral Health; Rega Institute for Medical Research.

[6] Calvo C., García López-Hortelano M., de Carlos Vicente J.C., Vázquez Martínez J.L. (2020). Recommendations on the clinical management of the COVID-19 infection by the «new coronavirus» SARS-CoV2. Spanish Paediatric Association working group. An. Pediatr..

[7] Zheng J. (2020). SARS-CoV-2: An Emerging Coronavirus that Causes a Global Threat. Int. J. Biol. Sci..

[8] The True Death Toll of COVID-19. Estimating Global Excess Mortality 20 May 2021. https://www.who.int/data/stories/the-true-death-toll-of-covid-19-estimating-global-excess-mortality.

[9] Legnani P., Checchi L., Pelliccioni G.A., D’Achille C. (1994). Atmospheric contamination during dental procedures. Quintessence Int..

[10] Timmerman M.F., Menso L., Steinfort J., van Winkelhoff A.J., van der Weijden G.A. (2004). Atmospheric contamination during ultrasonic scaling. J. Clin. Periodontol..

[11] Jain M., Mathur A., Mathur A., Mukhi P., Ahire M., Pingal C. (2020). Qualitative and quantitative analysis of bacterial aerosols in dental clinical settings: Risk exposure towards dentist, auxiliary staff, and patients. J. Fam. Med. Prim. Care.

[12] Chen I.-H., Lin C.-H., Liao Y.-S., Yang P.-W., Jao Y.-T., Chen T.-E., Du J.-K. (2022). Assessment of dental personal protective equipment (PPE) and the relationship between manual dexterity and dissemination of aerosol and splatter during the COVID-19 pandemic. J. Dent. Sci..

[13] Tysiąc-Miśta M., Dziedzic A. (2020). The Attitudes and Professional Approaches of Dental Practitioners during the COVID-19 Outbreak in Poland: A Cross-Sectional Survey. Int. J. Environ. Res. Public Health.

[14] Nasr L., Saleh N., Hleyhel M., El-Outa A., Noujeim Z. (2021). Acceptance of COVID-19 vaccination and its determinants among Lebanese dentists: A cross-sectional study. BMC Oral Health.

[15] Schmidt J., Perina V., Treglerova J., Pilbauerova N., Suchanek J., Smucler R. (2022). COVID-19 Vaccination among Czech Dentists. Vaccines.

[16] Lin G.S.S., Lee H.Y., Leong J.Z., Sulaiman M.M., Loo W.F., Tan W.W. (2022). COVID-19 vaccination acceptance among dental students and dental practitioners: A systematic review and meta-analysis. PLoS ONE.

[17] OECD/European Observatory on Health Systems and Policies (2023). Romania: Country Health Profile 2023. State of Health in the EU.

[18] World Health Organisation (WHO) (2021). WHO Coronavirus (COVID-19) Dashboard|WHO Coronavirus (COVID-19) Dashboard with Vaccination Data. https://covid19.who.int/.

[19] European Centre for Disease Prevention and Control (2020). COVID-19. https://www.europa.eu.

[20] Centers for Disease Control and Prevention (2020). Guidance for Dental Settings|CDC. CDC’s Updated Guidance for Dental Settings Addresses Screening, Infection Control Protocols—CDA. https://www.cdc.gov/coronavirus/2019-ncov/hcp/dental-settings.html.

[21] SCIENSANO (2020). Maitrise des Infections à SARS-VOV-2 Pour la Pratique Dentaire. https://covid-19.sciensano.be/sites/default/files/Covid19/COVID19_procedure_dentists_FR.pdf.

[22] Carvalho J.C., Declerck D., Jacquet W., Bottenberg P. (2021). Dentist Related Factors Associated with Implementation of COVID-19 Protective Measures: A National Survey. Int. J. Environ. Res. Public Health.

[23] World Health Organisation (WHO) (2020). Rational Use of Personal Protective Equipment for Coronavirus Disease (COVID-19) and Considerations during Severe Shortages. Interim Guidance. https://www.WHO-2019-nCov-IPC_PPE_use-2020.3-eng.pdf.

[24] Schwendicke F., Krois J., Gomez J. (2020). Impact of SARS-CoV2 (Covid-19) on dental practices: Economic analysis. J. Dent..

[25] Busnatu Ș.S., Niculescu A.G., Bolocan A., Andronic O., Pantea Stoian A.M., Scafa-Udriște A., Stănescu A.M.A., Păduraru D.N., Nicolescu M.I., Grumezescu A.M. (2022). A Review of Digital Health and Biotelemetry: Modern Approaches towards Personalized Medicine and Remote Health Assessment. J. Pers. Med..

[26] Maret D., Peters O.A., Vaysse F., Vigarios E. (2020). Integration of telemedicine into the public health response to COVID-19 must include dentists. Int. Endod. J..

[27] Meng L., Hua F., Bian Z. (2020). Coronavirus Disease 2019 (COVID-19): Emerging and Future Challenges for Dental and Oral Medicine. J. Dent. Res..

[28] Amato A., Caggiano M., Amato M., Moccia G., Capunzo M., De Caro F. (2020). Infection Control in Dental Practice During the COVID-19 Pandemic. Int. J. Environ. Res. Public Health.

[29] Romano M.R., Montericcio A., Montalbano C., Raimondi R., Allegrini D., Ricciardelli G., Angi M., Pagano L., Romano V. (2020). Facing COVID-19 in ophthalmology department. Curr. Eye Res..

[30] Howard-Reed C., Wallace L.A., Ott W.R. (2002). The effect of opening windows on air change rates in two homes. J. Air Waste Manag. Assoc..

[31] Plaza-Ruiz S.P., Barbosa-Liz D.M., Agudelo-Suárez A.A. (2021). Ventilation and air-conditioning systems in dental clinics and COVID-19: How much do we know?. J. Clin. Exp. Dent..

[32] Passarelli P.C., Rella E., Manicone P.F., Garcia-Godoy F., D’Addona A. (2020). The impact of the COVID-19 infection in dentistry. Exp. Biol. Med..

[33] Săndulescu M., Nicolescu M.I., Funieru C., Şahin G.Ö., Săndulescu O., ESCMID Study Group for Viral Hepatitis (ESGVH) (2023). Exposure to Biological Fluids in Dental Practice-Narrative Review on Appropriate Risk Assessment to Guide Post-Exposure Management. Pathogens.

[34] Garus-Pakowska A., Górajski M., Szatko F. (2017). Knowledge and Attitudes of Dentists with Respect to the Risks of Blood-Borne Pathogens-A Cross-Sectional Study in Poland. Int. J. Environ. Res. Public Health.

[35] Jain V.M., Parihar S.R.S., Acharya S., Acharya S. (2023). Effects of wearing personal protective equipment (PPE) and its role in affecting the work efficiency of dentists during the COVID-19 pandemic. Work.

[36] Caggiano M., Acerra A., Martina S., Galdi M., D’Ambrosio F. (2023). Infection Control in Dental Practice during the COVID-19 Pandemic: What Is Changed?. Int. J. Environ. Res. Public Health.

